# Addressing unique challenges and crafting inclusive policies for Queer living with disabilities

**DOI:** 10.4102/ajod.v13i0.1418

**Published:** 2024-09-30

**Authors:** Ikekhwa A. Ikhile, Azwihangwisi H. Mavhandu-Mudzusi, Ndlovu Sinegugu

**Affiliations:** 1 Department of Gender and Sexuality Studies, College of Human Sciences, University of South Africa, Pretoria, South Africa; 2 College of Human Sciences, University of South Africa, Pretoria, South Africa

**Keywords:** intersectionality, queer individuals living with disabilities, inclusive, challenges, policies

## Abstract

**Background:**

This article addresses the intersectional challenges faced by Queer people living with disabilities (QPwD).

**Objectives:**

The article aims to highlight the nature and extent of their struggles while proposing inclusive policies for societal integration and equality. Amid global efforts to promote Queer rights, discrimination and violence persist, particularly affecting those with disabilities.

**Method:**

Drawing upon a comprehensive review of literature and empirical research, this study investigated the experiences of QPwD, identifying key challenges such as limited access to inclusive services, heightened vulnerability to abuse and systemic unemployment. The methodological approach used in this study involves synthesising existing scholarship and empirical evidence to inform the proposed inclusive policies.

**Results:**

The findings reveal pervasive barriers encountered by QPwD, including societal stigma, attitudinal biases and physical obstacles. Discrimination in employment, education and healthcare further exacerbates their marginalisation, necessitating proactive measures to address systemic inequalities and promote social inclusion.

**Conclusion:**

In conclusion, this article underscores the urgent need for policy reforms and societal interventions to uphold the rights and dignity of QPwD. By advocating for Queer-inclusive workplace policies, enhancing accessibility in public infrastructure and fostering governmental support for equality initiatives, this study contributes to advancing social justice and inclusivity.

**Contribution:**

The article calls for concerted efforts to create a more equitable and inclusive society where all individuals, regardless of their sexual orientation or disability status, can thrive and fulfil their potential.

## Introduction

While the exploration of intersectional identities is increasing, there remains a scarcity of published studies examining the experiences of individuals who identify as Lesbian, Gay, Bisexual, Transgender, Intersex and Questioning (LGBTIQ) and live with disabilities. In this article, we will refer to this population as Queer people living with disabilities (QPwD). This study is a qualitative investigation utilising secondary data to explore the personal experiences of persons who identify as members of the QPwD. The study examines numerous aspects of the lives of individuals with disabilities, including their perspectives, experiences with discrimination, educational and career aspirations, financial problems, physical and psychological health, social support networks and intimate relationships. This analysis is based on a comparison of multiple secondary research findings.

To contextualise our examination, it was imperative to delineate the overarching challenges and encounters faced by the study population. Modern society mostly follows norms that favour people who are heterosexual and physically able. This means that anyone who does not fit these norms, including those with different sexual orientations, gender identities or people without disabilities, often face being left out or treated unfairly (Chappell 2015; Egner 2019; Wellard 2006).

Within this paradigm, individuals identifying as QPwD find themselves situated within stigmatised minority groups (Miller 2018). The confluence of these intersecting identities amplifies the experiences of marginalisation and discrimination encountered within both Queer and disability communities.

Therefore, comprehending the intricate tapestry of experiences among this target population necessitated an exploration of intersectionality, acceptance dynamics within Queer and disability spheres and the journey towards self-acceptance and identity actualisation. By embracing the complexities of societal norms and individual identities, scholars and practitioners can work towards cultivating more inclusive environments and robust support systems for QPwD.

Individuals with disabilities encompass a diverse spectrum of sexual orientations, spanning from heterosexual to queer identities; yet, their experiences within the Queer community often remain overlooked and marginalised (Ramasamy, Rillotta & Alexander 2021). This nuanced intersectionality intertwines common challenges encountered by the broader Queer population, including bullying, abuse and the complexities of navigating acceptance amid societal heteronormativity and ableism (Smilges 2022).

Bullying, a pervasive issue among individuals with disabilities, often intersects with experiences related to sexuality and gender expression, manifesting in verbal abuse, threats of violence and physical assault (Dinwoodie, Greenhill & Cookson 2020). Such maltreatment perpetuates stigmatisation rooted in ableism and heteronormative assumptions, exacerbating segregation and impeding access to fulfilling life opportunities (Tan & Saw 2023). This culture of exclusion reverberates across public spaces, including governmental and non-governmental organisations tasked with serving individuals with disabilities (Tan & Saw 2023).

Acknowledging the importance of safe spaces, Queer support groups emerge as vital institution where individuals with disabilities can explore their identities, foster connections and integrate into supportive communities (Bates 2018). Lewis and Herman’s (2022) study underscores the transformative impact of support groups, illuminating how such environments empower individuals to cultivate self-affirmation and extend support to others, thereby nurturing resilience and collective solidarity.

In synthesising these dimensions of intersectionality, it becomes evident that the experiences of QPwD are multifaceted and demand nuanced policy responses and social interventions. By amplifying the voices of marginalised communities and fostering inclusive environments, we endeavour to dismantle systemic barriers and cultivate a society where diversity is celebrated, and all individuals, irrespective of their abilities or sexual orientations, are afforded dignity, respect and equitable opportunities for fulfilment.

## Lesbian, Gay, Bisexual, Transgender, Intersex and Questioning+ study context

The acronym ‘LGBTQI+’ (Lesbian, Gay, Bisexual, Transgender, Questioning, Intersex Plus) is a condensed form of commonly used terms that pertain to sexual orientation and gender identity. The investigation of gender and sexual orientation may be historically linked to the 1950s, when the labels ‘lesbian’ and ‘gay’ were coined to refer to individuals who do not identify as heterosexual. Subsequently, the terms ‘bisexual’, ‘transgender’, ‘queer’ and ‘intersex’ were included in the acronym with the purpose of promoting opposition to homophobia. The plus sign denotes the incorporation of additional non-binary distinguishing identities that are not explicitly mentioned in the abbreviation. Despite the promotion of diversity in academic discourses around gender and sexual orientation, stigma and prejudice continue to exist within the LGBTQI+ community. Mavhandu-Mudzusi et al. (2023) conducted a study to determine the preferred terminology for addressing LGBTQI+ individuals, with ‘Queer’ emerging as the majority preference. As a result, the specific population is referred to as QPwD in this article.

### Disability study context

The United Nation Convention on the Rights of Persons with Disabilities defines disability as long-term physical, mental, intellectual or sensory impairments (Guide 2014). When combined with additional difficulties, these limitations may prevent people from participating fully and equally in society. Disability comprises several conditions, each with its own issues and peculiarities, creating various disability communities. Based on this study, the secondary research data gathered focusses on individuals with any physical disabilities who identify as Queer.

### Intersectionality in a South African context

In the South African context, intersectionality illuminates the intricate dynamics of belonging to multiple identities and social groups (Maxwell et al. 2016). Rooted in black feminism, intersectionality underscores how various forms of oppression, including gender, sexual orientation, race, religion, national origin and class, intersect to shape the experiences of individuals within marginalised communities (Nash 2011). Collins and Bilge (2020) emphasise the significance of examining intersectionality not only in terms of its impact on individual self-perception but also in understanding its role in perpetuating power imbalances and inequalities.

Being situated at the intersection of multiple stigmatised identities, unveils the challenges of deviating from normative standards and navigating layered oppression. South Africa QPwD encounter multiple systems of inequality, such as ableism, homophobia, heterosexism, classism, racism and ageism (Richardson & Monro 2017). For example, lesbian women with disabilities may face negative attitudes regarding both their sexual orientation and disabilities, leading to compounded stigmatisation and oppression. Queer People with Disability confront ableist and heteronormative societal perceptions that often deny or restrict access to their sexual identities. It therefore becomes imperative to comprehend the experiences of QPwD through an intersectional lens to address their unique challenges and needs within the South African context (Kempapidis et al. 2023).

Moreover, negotiating one’s position within both the Queer and disability communities pose an additional challenge. Individuals with disabilities who identify as Queer may struggle to find a sense of belonging within either group, experiencing homophobia within the disability community and disablism within the Queer community (Leonard & Mann 2018). Similarly, individuals who are deaf and identify as Queer may encounter unique struggles as an ‘invisible’ minority, navigating the complex intersections between the Queer and disability communities (Miller & Clark 2019).

Drawing upon the concept of intersectionality in South Africa, researchers advocate for centring the voices of marginalised individuals and analysing the interplay between individual experiences and larger systems of power and privilege (Van Herk, Smith & Andrew 2011). Intersectional studies must encompass micro-level experiences while analysing the influence of macrolevel factors such as systems of power and privilege (Bayrakdar & King 2023). Embracing intersectionality facilitates a deeper understanding of how identities intersect and interact within the South African context, guiding efforts towards social justice and inclusivity.

### Queer people living with disabilities sexuality experiences

In South Africa, QPwD face a multifaceted landscape shaped by intersecting identities and systemic challenges (Msekele 2020). While significant strides have been made in Queer rights, including the legalisation of same-sex marriage and constitutional protections against discrimination based on sexual orientation, marginalised communities continue to grapple with entrenched prejudices and structural barriers unique to the South African context.

Boonzaier and Mkhize (2018) affirm that QPwD struggle with navigating the complexities of disclosure and social integration within South Africa’s higher education institutions. Miller (2018) underscore the challenges of stigma and discrimination, exacerbated by a lack of inclusive policies and support mechanisms. Boonzaier and Mkhize (2018) further highlight that despite legislative progress, Queer students with disabilities often face invisibility and marginalisation, hindering their academic and personal development.

The cultural backdrop of South Africa reflects enduring norms of heteronormativity and ableism, perpetuating marginalisation and exclusion within disability organisations and institutional settings (Smith et al. 2021). Despite legal protections, QPwD encounter systemic barriers limiting access to essential services and impede social participation (Van der Heijden et al. 2020). Trans individuals with intellectual disabilities face unique challenges in accessing healthcare and community support, exacerbating feelings of vulnerability and isolation (Smith et al. 2021).

In response, QPwD demonstrate resilience and agency, challenging societal norms and advocating for recognition of their rights and dignity. Initiatives such as Queer support groups and advocacy organisations provide vital safe spaces for identity exploration and community-building (Bates 2020).

The experiences of QPwD underscore the imperative of intersectional advocacy and policy reform. South Africa’s National Development Plan recognises the need for inclusive policies and interventions to address the marginalisation faced by Queer individuals. However, implementation gaps and institutional inertia pose significant challenges to realising the plan’s vision of a more inclusive society (Dalvit 2022; National Planning Commission 2012).

Moreover, South Africa’s legal framework for Queer rights remains unevenly implemented and subject to political and cultural contestation (Lewis 2021). While landmark judgements, such as the Constitutional Court’s ruling legalising same-sex marriage, signify progress, Queer individuals continue to face discrimination and violence in various spheres of life (Mkhize & Bennett 2020). The experiences of Queer individuals with disabilities in South Africa highlight the urgent need for intersectional advocacy, policy reform and social transformation. By confronting systemic barriers and fostering inclusive environments, South Africa can strive towards a society that embraces diversity, celebrates authenticity and ensures equitable opportunities for all its members.

### Queer people living with disabilities social support experiences

Navigating the complex intersection of individuals who are Queer living with disability, encounter varying degrees of social support that significantly influence their sense of belonging and well-being (Drummond & Brotman 2014). Within South Africa’s diverse social landscape, the dynamics of social support for QPwD reflect broader societal attitudes and institutional frameworks.

People with intellectual disabilities often grapple with labelling their identities, yet the acceptance and support offered by social groups and support networks can foster discussions around positive aspects of identity and mitigate negative beliefs (Tallentire et al. 2020). In South Africa, initiatives such as Queer support groups and community organisations play a pivotal role in providing safe spaces for individuals to explore their identities and build supportive networks (Bates 2020).

While digital gadgets hold promise for enhancing social involvement and sense of belonging among individuals with disabilities, significant challenges persist in their accessibility and usability (Dinwoodie et al. 2020). In a South African context, disparities in access to technology exacerbate existing inequalities, particularly for QPwD residing in marginalised communities.

The cancellation of Pride events in the wake of the coronavirus disease 2019 (COVID-19) pandemic underscored the profound impact on QPwD, who experienced heightened challenges compared to their counterparts without disabilities (Lewis et al. 2017). For many, Pride events serve as vital platforms for visibility, community engagement and advocacy, fostering a sense of belonging and empowerment. The absence of such events further marginalised QPwD, underscoring the importance of inclusive strategies in event planning and community organising. Moreover, making use of the online channel, such as the virtual Pride events, webinars and social media channels, can help to bridge the width created by the cancellation of the physical events. These digital platforms provide QPwDs access to alternative spaces for participation, networking and activism, in order to ensure that they remain included and empowered within the LGBTQ+ community despite the imposed social barriers caused by the pandemic (Ceia, Nothwehr & Wagner 2021).

The intersection of QPwD within South Africa’s social fabric reflects broader dynamics of acceptance, visibility and inclusion. Despite progress in Queer rights, significant gaps remain in ensuring equitable access to social support and community resources for individuals with disabilities. The social model of disability, which emphasises the role of societal barriers in limiting participation and inclusion, underscores the imperative of dismantling systemic inequalities and fostering inclusive environments (Oliver 2013).

## Research methods and design

This study uses a methodology that involves reviewing secondary articles for an exhaustive examination of the routine life of the QPwD in South Africa. The methodology section of the article outlines the approach taken to conduct a review of literature focussing on the daily experiences of adults in South Africa who identify as QPwD. The following elements were considered in the methodology:

### Conceptual research approach and rationale

A scoping review approach was adopted to comprehensively explore the existing literature on the experiences of adults in South Africa who identify as QPwD. This approach was chosen to provide a broad overview of the topic, identify key themes and uncover gaps in the literature.

### Inclusion and exclusion criteria

In an attempt to maintain relevance and coherency, the researchers had set out certain principles regarding the inclusion and exclusion of literature on QPwD experiences. These criteria were intended to match our research goals, specifying the inclusion of only the articles that could provide a direct insight into the subject matter. Following Meline’s (2006) direction, this approach ensures that the scope and research are kept on track.

#### Inclusion criteria

The study consists of social scientific data that have directly addressed the daily life of the QPwD. These findings would cover the different areas of social life, such as health, well-being, employment, education, discrimination, support and intimate relations.

#### Exclusion criteria

In order to be relevant to the current legislative state as well as social expectations regarding disability and sexual diversity, articles published before the year 2000 were excluded from consideration. Also, articles not in English were dropped because of the language barrier issue. Pieces of text that are not available in the whole-text form were also not taken into consideration to make the analysis complete. Moreover, articles that addressed only intellectual disabilities were eliminated in order to maintain the focus on the experiences of QPwD across a wide range of disabilities.

### Systematic review process for secondary data

On 05 February 2024, the researchers concluded the systematic review process as the secondary data. The authors employed the framework developed by Arksey and O’Malley (2005) as a reference to conduct a thorough search among the existing literature. This involved a search in established academic databases relevant to both South Africa and global perspectives, such as Sabinet, SciELO South Africa, African Journals Online (AJOL), the National Electronic Library of South Africa (NELSON), PubMed, Embase, PsycInfo and Social Policy and Practice. Furthermore, the researcher combined the Boolean search technique, which was suggested by Aliyu (2017), to increase the precision and quality of the literature review process. Initially, 210 records were screened by a research assistant based on abstracts and full text. Next, after the removal of duplicates and after applying the eligibility criteria a total of 82 articles were excluded. Generally, the strategy entailed the use of a scientific system to identify and analyse literature about the life experiences of QPwD in South Africa and other parts of the world for a deeper understanding of the issue at hand.

**FIGURE 1:**
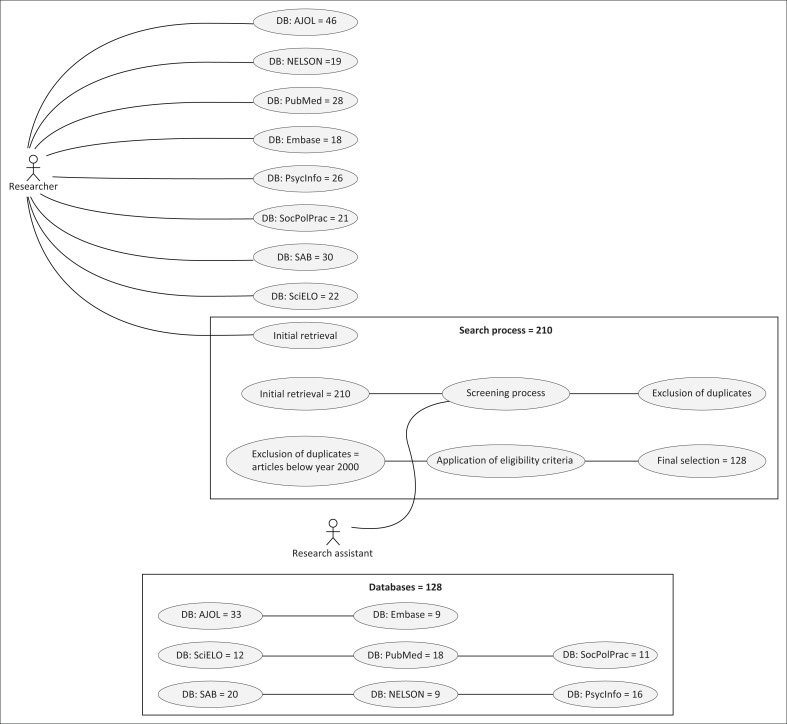
Selection process steps.

For the search and selection process, 128 articles were left, out of which the main domains included health, well-being, employment, education, discrimination, support and intimate relationships of QPwD (Brakewood & Poldrack 2013). However, the major part of our review focussed on physical disabilities but is definitely aware of the diversity of disabilities and complexities in their experience. Despite the fact that certain studies had involved Queer individuals with intellectual disabilities, our specialisation was still on physical disabilities. Furthermore, we accessed grey literature to dig deeper into the subject through searching of government and charity reports. More information about the process is elaborated in the Figure 1.

### Thematic analysis

The method used in this study to perform thematic analysis included going through the selected articles and extracting patterns and themes concerning the QPwD participation experiences following the guidelines outlined by Nowell et al. (2017). As a start, we classified the articles based on the main themes, including social support experiences, sexuality experiences and others. From these groupings, it is possible to identify some commonalities and differences across the studies. This process enabled us to come up with wider concepts that would capture the various challenges facing QPwD. After several debates and revisions, the members of the research team agreed on the major themes that captured the essential evidence presented in the literature. These themes are as shown in Table 1.

**TABLE 1:** Superordinate themes.

Superordinate themes	Key findings areas
Double Marginalisation	QPwD’s experiences with stigmatisation and discrimination based on disability and sexuality.
Limited Access to Inclusive Services	Inclusive and non-inclusive access barriers for QPwD in healthcare, education, transport, and social services.
Bullying and Exclusion	Cases when QPwD experience bullying, harassment or social ostracism.
Economic Insecurity and Employment Discrimination	Occupation barriers faced by QPwD because of job opportunities and economic insecurity.
Intersectional Discrimination in Healthcare	Intersectional discrimination: Compounded barriers in healthcare settings for QPwD.
Legal Framework	Legal protections and policy gaps which affect QPwD based on their disability and sexuality.

QPwD, Queer people living with disabilities.

### Ethical considerations

This article followed all ethical standards for research without direct contact with human or animal subjects. The article still abides by the ethical principles, which was to justify the referencing and acknowledgement of all sources that were used in the review process (Brakewood & Poldrack 2013).

## Results

This study clarifies the complex and sometimes overlooked issues encountered by South African QPwD (Smith 2023). The intersections of sexual orientation, gender identity and disability status were explored to reveal QPwD’s specific discrimination, exclusion and marginalisation (Hunt et al. 2006). The findings show that QPwDs face double marginalisation, with additional impediments to social inclusion, economic security and access to key resources (Johnson & Patel 2021). Queer people with disability encounter many obstacles that make them more vulnerable to discrimination and social marginalisation, from restricted access to inclusive healthcare and support services to increased school bullying and exclusion (Brown & Garcia 2020). The study also shows systemic gaps in legal and policy frameworks protecting QPwD’s rights and protections, emphasising the need for more comprehensive and intersectional approaches to address their unique needs and promote equity and social inclusion (Gupta & Lee 2019). This study analyses data to inform inclusive policies and initiatives aimed at enhancing support for QPwD in South Africa and internationally (Choudhury, Blakemore & Charman 2006).

### Double marginalisation

The study population both in South Africa and other countries encounter additional difficulties because of the overlap of their sexual orientation, gender identity and disability status, which heighten their susceptibility to discrimination, exclusion and violence (Bulter & Faucault 2009). The research conducted by Mafumo (2011) emphasises that persons who possess several marginalisation identities, such as QPwD, face distinct obstacles that cross and amplify, resulting in increased instances of discrimination and exclusion. For example, a Queer person with disability may encounter stigma and discrimination as a Queer individual and structural obstacles while trying to access healthcare services that cater to their varied needs as someone living with disability (Lee & Kanji 2017). Moreover, Conde’s research (2018) highlights the heightened vulnerability of QPwD to hate-motivated violence and harassment as a result of the convergence of their sexual identities.

Msekele (2020), in her research article called ‘The Blind Gaze’, vividly recounts the harrowing tale of a young woman named Zee, who courageously navigates life as a Queer individual living with a disability. Zee’s story is one of resilience and adversity, marked by the profound challenges she has faced. Tragically, Zee was subjected to the horrors of sexual assault not once, but twice, each instance leaving an indelible mark on her life’s trajectory.

The first assault occurred when Zee was a vulnerable disabled girl, targeted by perpetrators who preyed upon her physical limitations and perceived vulnerabilities. The second traumatic incident occurred later in her life, when Zee was targeted specifically for her identity as a Queer woman. This dual victimisation underscores the intersecting layers of discrimination and violence faced by individuals such as Zee, who navigate the complex terrain of disability and sexual orientation (Daigle et al. 2024). In the context of South Africa, where Zee resides, the scourge of corrective rape looms large, particularly for Queer women. This reprehensible practice, fuelled by bigotry and prejudice, seeks to ‘correct’ or punish individuals for their sexual orientation or gender identity. It represents a grave violation of human rights and a stark reminder of the pervasive homophobia and transphobia that persist in society.

Moreover, Zee’s story sheds light on another troubling phenomenon: the fetishisation and objectification of disabled individuals for sexual gratification. Some individuals derive perverse pleasure from fantasising about engaging in sexual acts with disabled women, perpetuating harmful stereotypes and perpetuating the exploitation of vulnerable individuals (Kim 2014). Zee’s narrative serves as a poignant reminder of the urgent need for comprehensive societal awareness, education and advocacy to combat the scourge of sexual violence and discrimination against marginalisation of communities. It underscores the imperative of fostering a culture of empathy, respect and inclusivity, where all individuals are valued and their rights upheld without exception. Through collective action and solidarity, we can strive to create a world where stories such as Zee’s are not only heard, but met with compassion, justice and support.

Trani et al.’s (2020) research underscores the pervasiveness of prejudice and bias faced by individuals who identify as both disabled and LGBTQ+. This leads to an increased susceptibility to mental health difficulties and exclusion from society. Furthermore, Casey et al.’s (2019) research highlights the dual, systematic discrimination that QPwD encounter, resulting in significantly elevated rates of unemployment and economic instability. Sherry (2004) also recognises the overlapping challenges experienced by QPwD in her research, affirming that difficulties confront universalising norms that marginalise the study population.

This study emphasises the immediate requirement for comprehensive and intersectional strategies to tackle the distinct needs experience by QPwD. Policy measures and interventions should be based on a comprehensive understanding of the various forms of marginalisation faced by QPwD (Zeeman et al. 2019). Mulé et al. (2009) further affirm that these measures should prioritise inclusive practices that acknowledge and uphold the dignity and rights of all individuals, irrespective of their intersecting identities. Efforts to address prejudice and promote inclusivity must adopt an intersectional approach, acknowledging the interconnectedness of various forms of oppression and striving to dismantle discriminatory structures that perpetuate marginalisation for individuals who identify as queer, disabled or both (Thomas et al. 2021).

### Limited access to inclusive and non-inclusive services

Queer individuals who have disabilities face significant obstacles when trying to access services that are both Queer-inclusive and completely accessible, especially in the rural areas of South Africa (Hunt et al. 2021). The study conducted by Nkosi and Molebatsi (2019) emphasises the significant inequalities in healthcare availability experienced by people living with disability residing in rural areas in South Africa, which are further exacerbated by their sexuality status. The geographic isolation of these areas restricts the access to healthcare facilities and support services, making QPwD particularly susceptible to discrimination and social exclusion (Lee & Robert 2017).

Furthermore, research conducted by McKinney and Swartz (2020) highlights the widespread discrimination and limited understanding regarding the healthcare requirements of QPwD in South Africa. This worsens their difficulties in obtaining suitable care and assistance. These findings emphasise the immediate need for specific governmental interventions and community-led initiatives that aim to improve the availability and inclusiveness of services for QPwD throughout South Africa, especially in rural and underserved regions (Bragazzi et al. 2023). Effective cooperation between government agencies, healthcare providers, advocacy organisations, civil society organisations (CSOs) and local communities is crucial in tackling these structural obstacles and advancing fair and equal access to vital services for all individuals in the Queer community, regardless of their disability status.

### Heightened bullying and exclusion

Reygan, Henderson and Khan (2022) affirm that bullying and marginalisation are major, widespread issues faced by QPwD across South Africa, with the educational sector being one of the most impacted. Studies reveal significantly elevated levels of harassment and bullying encountered by QPwD individuals, who are subject to a greater extent of harassment and bullying than their non-disabled counterparts (Commission for Gender Equality 2015; Mafumo 2011). This concerning pattern highlights the immediate necessity to tackle structural problems that contribute to the exclusion of young individuals with disabilities from educational environments. Younger QPwD in South Africa face substantial obstacles to achieving social integration and academic achievement as a result of the widespread occurrence of bullying and harassment (Mafumo 2011). Mafumo’s study also emphasises the alarming fact that those with disabilities are more vulnerable to encountering bullying and harassment in comparison to those without disabilities. The ongoing victimisation experienced by students with disabilities contributes to an environment of exclusion and alienation, compromising their sense of safety and well-being within educational institutions. Furthermore, the consequences of bullying and exclusion for youth with disabilities go beyond the classroom setting and frequently lead to negative educational results (Mafumo 2011). The increased susceptibility of youth with disabilities to bullying and exclusion worsens their sense of social isolation and hinders their scholastic advancement, thus reinforcing cycles of marginalisation and inequity.

To effectively tackle the systemic issues of bullying and exclusion faced by Queer youth with disability, it is necessary to implement comprehensive interventions that give priority to inclusivity and fairness in educational settings (National Council of Provinces 2015). Young, Ne’eman and Gelser (2011) highlight the importance for South African politicians and educational stakeholders to give top priority to creating and enforcing thorough anti-bullying policies and providing appropriate support systems specifically designed for children with disabilities and other conditions. In addition, promoting a culture of diversity and acceptance in schools through focussed awareness campaigns and inclusive curricula can reduce the occurrence of bullying and create a more inclusive learning environment for all students, regardless of their sexual orientation or disability status (Yell et al. 2016). To create a more inclusive educational environment in South Africa, it is important to recognise the intersectionality of Queer identity and disability (Reygan et al. 2022). By actively addressing the systemic obstacles that Queer youth with disability encounter, government through the basic and higher educational departments should promote a safe and supportive space where all students have the opportunity to learn and succeed, while also respecting their dignity and rights.

### Economic insecurity and employment discrimination

Studies have shown that QPwDs in South Africa have significantly higher levels of joblessness and face discrimination in the workplace, which worsens their financial instability and perpetuates patterns of exclusion (Casey et al. 2019; National Council of Provinces 2017):

They denied me of the position I qualified for because I was black, disabled and a queer family. Reygan et al. (2022)

The preceding quote exemplifies the deep-seated intersectional discrimination encountered by an individual, who was denied a position they were qualified for based on their race, disability and sexual orientation. This highlights the structural obstacles present in job sectors where there is a convergence of discrimination based on race, disability and sexual orientation. This individual’s experience underscores the imperative for implementing comprehensive anti-discrimination rules and inclusive practices in workplaces to guarantee equitable opportunities for all persons, irrespective of their ethnicity, disability status or sexual orientation. This form of discrimination not only denies individuals their deserved rights and access but also shows continuous patterns of marginalisation and exclusion, impeding progress towards achieving diversity, fairness and inclusivity in our society (Steyn et al. 2020).

Several studies have revealed significant discrepancies in employment rates between individuals with disabilities and those without, underscoring structural obstacles that impede the access of qualified QPwD to meaningful job prospects (Casey et al. 2019; Clare 2015; Meyer 2003; Müller & Daskilewicz 2018; Porter 2023; Samuels 2003). Some barriers to equal opportunities for qualified QPwD in accessing meaningful job prospect in South Africa are a lack of access ramps in business and transportation facilities (Ned & Lorenzo 2016). Discrimination at workplaces and unfair treatment of employees with disability and negative attitudes towards disability and genderism are still a reality (Marumoagae 2012). Thus, the problem is worsened by the low rate of employment equity legislation implementation (Oosthuizen & Naidoo 2010). Furthermore, inadequate accessible education and few vocational programmes hinder skills acquisition (Mutanga 2017). In the South African context, disability is often combined with other forms of oppression, including race and gender, which exacerbates these barriers (Moodley & Graham 2015). Discriminatory practices towards marginalised individuals with disabilities, different sexual orientations, and diverse gender identities can never be over emphasised (National Council of Provinces 2017). The study conducted by Garofalo (2011) emphasises the widespread occurrence of workplace discrimination faced by individuals with disabilities, emphasising the necessity of comprehensive governmental measures to tackle systemic inequalities.

Coulter-Thompson et al. (2023) also affirmed that the combination of disability and Queer identity in South Africa creates additional difficulties for QPwD when it comes to accessing jobs and support services. The presence of discriminatory employment practices and workplace conditions escalate obstacles to economic involvement for individuals with disabilities, hence increasing their susceptibility to poverty and social exclusion (Garofalo 2011).

In order to tackle the widespread issues of unemployment and employment discrimination faced by QPwD, it is crucial to make focussed and collaborative endeavours to encourage inclusive recruitment practices, deconstruct discriminatory laws and improve the availability of vocational training and support services (Carew et al. 2020). In progressing towards a more equitable and inclusive society, South Africa can prioritise the economic empowerment of QPwD and promote inclusive workplaces that acknowledge and accept varied abilities and identities (McKinney Desposito & Yoon 2020).

### Intersectional discrimination in healthcare

The struggle against structural barriers that hinder better access to health services for the Queer community in South Africa is still a reality (Daigle et al. 2024). Queer people living with disability in South Africa encounter substantial challenges in accessing inclusive and competent healthcare services, exacerbating existing health disparities and perpetuating cycles of marginalisation (Rohleder et al. 2018; Watermeyer et al. 2018). Discriminatory practices and attitudes within healthcare settings further marginalise QPwD, hindering their access to quality healthcare and support services (Watermeyer et al. 2018). Research indicates that QPwD often face neglect of their specific healthcare needs because of the intersectionality of Queer identity and disability (Rohleder et al. 2018). This neglect exacerbates health disparities and contributes to the disproportionate burden of illness experienced by QPwD (Watermeyer et al. 2018). Additionally, stigma and discrimination within healthcare settings deter QPwD from seeking timely and appropriate care, further exacerbating health outcomes (Rohleder et al. 2018).

The intersectional nature of Queer identity and disability underscores the need for tailored and inclusive healthcare services that address the diverse needs and experiences of QPwD (Watermeyer et al. 2018). Comprehensive training programmes for healthcare providers on LGBTQ+ cultural competency and disability awareness are essential to mitigate discriminatory practices and improve the accessibility of healthcare services for QPwD (Rohleder et al. 2018).

Furthermore, policy interventions aimed at promoting the rights and inclusion of QPwD in healthcare settings are imperative to address systemic barriers and ensure equitable access to healthcare services (Watermeyer et al. 2018). By prioritising the development of inclusive healthcare policies and fostering environments that respect and accommodate diverse identities and abilities, South Africa can advance towards a more equitable and inclusive healthcare system.

### Legal and policy frameworks

The study revealed substantial gaps in the existing legal and policy frameworks concerning the rights and safeguards of QPwD in South Africa (Davis 2019; Watermeyer et al. 2018). Although there are laws in existence to combat discrimination based on sexual orientation, gender identity and handicap status, their effectiveness is hindered by gaps in the enforcement mechanisms and implementation (Davis 2019).

Existing anti-discrimination laws are inadequate in protecting persons with disabilities from intersectional discrimination, putting the affected parties in a precarious position of exclusion (Watermeyer et al. 2018). A failure of legislation to provide special provisions for disability-related barriers perpetuates disparities as well as impacts the advancement of integration and equity for those with a disability (Davis 2019). Enhancing the legal frameworks and promotion of policy change are ways to combat the multiple layered discrimination experienced by persons with disabilities in South Africa (Watermeyer et al. 2018). It is hence important for effective implementation of existing laws and policies, to also have strong enforcement measures and prevention measures against systematic discrimination (Davis 2019). South Africa stands as one of the few countries in Africa where same-sex marriage has been legalised, yet Queer people continue to be discriminated against and stigmatised. These social attitudes greatly affect the adoption and acknowledgement of such policies aimed at enhancing their rights (Sichinga 2022). Government agencies, CSOs and disability and sexuality rights activists need to work together to advocate for the rights and needs of QPwD and spur policy development (Watermeyer et al. 2018). To work towards the transformation of South Africa to a more equal and just society, emphasis should be placed on the enhancement of legal and policy frameworks that will accommodate the multiple dimensions and needs of QPwD.

This study reveals important implications for recognising the imperativeness of diversified and comprehensive solutions to support QPwD who confront specific obstacles. It states that understanding multiple types of discrimination and exclusion will allow different interested parties within and outside the framework of institutions to come up with equitable and inclusive initiatives. Therefore, initiatives should strive at promoting and protecting the rights and human dignity of every person without discriminating against the lesbians, gays, bisexuals, transgender persons, and persons with disabilities. Key stakeholders in this process include:

- Healthcare professional and providers
- Civil society organisations
- Government and/or policymakers
- Educators
- Community leaders and members.

It is therefore important that these groups engage in collaborative action to bring about change and promote a more inclusive society for QPwD.

## Discussion

This study presents important recommendations derived from the various reviewed articles with a focus on QPwDs. The process followed a systematic review of literatures across academic databases, screening of articles and then developing a thematic analysis. Through this process, key hindering factors that impact negatively on QPwD were identified, which then gives room for the development of conforming policies. The recommendations in this study are supported by data and articulate the multifaceted needs of QPwD, thereby leading to improved inclusiveness and support within society. Key recommendation that were generated from the reviews based on the study findings are as follows:

### Policy reform for inclusive healthcare

As supported by Switzer (2003), policymakers must give priority to the creation and execution of healthcare policies that are inclusive and specifically cater to the distinct requirements of QPwD. This includes the promotion of accessibility to healthcare practitioners who are knowledgeable and skilled in LGBTQ+ healthcare, the education of healthcare professionals on inclusive approaches and the eradication of discriminatory practices within healthcare environments.

### Promotion of inclusive employment practices

Promoting inclusive employment practices is crucial to combat employment discrimination experienced by QPwD. Policymakers and businesses ought to enforce anti-discrimination policies, offer appropriate accommodations and cultivate inclusive workplace cultures that embrace diversity and advance fair opportunities for individuals with disabilities in the workforce (Priola et al. 2014).

### Social support networks and community engagement

Establishing community-based projects and support networks is crucial for providing social support and promoting inclusion for individuals with disabilities (Duggan & Linehan 2013). These programmes can enhance peer support, foster social connections and address the stigma and discrimination faced by those with disabilities in their communities.

### Education and awareness programmes

Education and awareness initiatives should be established to foster comprehension and embracing of intersectional identities, encompassing Queer and disability identities (Egner 2018). These programmes can be introduced in educational institutions, professional environments and community venues to confront preconceived notions, promote understanding and cultivate inclusive mindsets towards individuals with disabilities.

### Collaborative advocacy efforts

It is crucial to promote collaboration among Queer rights organisations, government, disability advocacy groups, policymakers and community stakeholders in advocacy endeavours. Through collaboration, these groups may enhance the influence of QPwD, champion policy reforms and facilitate structural transformations that support the rights and integration of QPwD in the society (Brubaker, Harper & Singh 2011).

### Research and data collection

Research and data collection are crucial for gaining a more profound comprehension of the complex issues faced by QPwD and for guiding policy and practice based on solid facts (Dinwoodie et al. 2020). Allocation of funding should prioritise research programmes that specifically address the experiences and needs of those who identify as QPwD, with an emphasis on incorporating various perspectives and real-life experiences.

### Empowerment and self-advocacy

Development of empowerment and self-advocacy programmes is necessary to provide QPwD with the necessary knowledge, skills, and resources to effectively advocate for their rights and fight against intersectional discrimination (Dowse 2001). These programmes offer specialised training in self-advocacy, leadership development and empowerment tactics that are customised to meet the specific requirements of those who identify as QPwD.

To foster greater inclusivity and fairness for queer individuals with disabilities, policymakers, advocacy groups, government, CSO and community stakeholders should adopt these essential suggestions. This will contribute to the promotion of social justice and the advancement of human rights for all individuals, irrespective of their overlapping identities.

## Conclusion

To summarise, this study provides insight into the overlapping difficulties encountered by QPwD in South Africa and emphasises the immediate requirement for focussed interventions to tackle their distinct needs and experiences. By examining factors such as healthcare accessibility, employment bias, school bullying, exclusion and legal gaps, it becomes clear that individuals with disabilities confront complex discrimination and marginalisation.

The findings expose structural obstacles that impede the social integration and welfare of QPwD, underscoring the necessity for extensive legislative overhauls and community-driven initiatives. To effectively tackle the various types of discrimination experienced by those with disabilities, a comprehensive strategy is needed that places emphasis on inclusiveness, fairness and societal fairness. Essential suggestions involve creating and executing comprehensive policies that acknowledge and tackle the overlapping identities and requirements of individuals with disabilities. Improving legal safeguards, expanding access to comprehensive healthcare treatments and advocating for inclusive educational settings are crucial measures for promoting social inclusion and equity for QPwD in South Africa.

Moreover, it is essential to have cooperative endeavours among government agencies, CSOs, and disability rights activists in order to champion the rights and interests of those with disabilities and to bring about significant policy transformation. To evolve towards a more inclusive and equitable society, South Africa can prioritise the establishment of comprehensive legal and policy frameworks that acknowledge and tackle the overlapping identities and needs of QPwD. This study highlights the significance of acknowledging and dealing with the overlapping types of discrimination experienced by QPwD in order to promote a society that respects the dignity, rights and welfare of all individuals, irrespective of their sexual orientation, gender identity or disability status. By implementing coordinated endeavours and fostering collective participation, South Africa has the potential to establish a path towards a future that is more inclusive and fairer for QPwD, thereby creating a society that embraces diversity and enables the flourishing of all individuals.
